# Evaluating the Efficacy of Electroacupuncture Compared to Physiotherapy in Reducing Pain and Disability in Soldiers Diagnosed with Chondromalacia Patella: A Randomized Clinical Trial

**DOI:** 10.5812/aapm-143688

**Published:** 2024-09-03

**Authors:** Reza Kazempour Mofrad, Afsaneh Dadarkhah, Zahra Rezasoltani, Sharif Najafi

**Affiliations:** 1 Trauma and Surgery Research Center, AJA University of Medical Sciences, Tehran, Iran; 2 Clinical Biomechanics and Ergonomics Research Center, AJA University of Medical Sciences, Tehran, Iran

**Keywords:** Electroacupuncture, Physical Therapy Modalities, Chondromalacia Patella, Anterior Knee Pain Syndrome

## Abstract

**Background:**

One of the most prevalent causes of anterior knee pain is chondromalacia patella (CMP), particularly among young populations, including soldiers. Although various treatments have been suggested to relieve the symptoms and improve the functioning of patients with CMP, none have proven to be adequately effective.

**Objectives:**

This study assessed the effectiveness of electroacupuncture as a complementary treatment for CMP, noting its minimal complications.

**Methods:**

In this randomized clinical trial, soldiers diagnosed with CMP who met the inclusion criteria and referred to Imam Reza Hospital in Tehran in 2023 were assigned to one of two treatment groups. The electroacupuncture group received treatment at specific acupuncture points combined with an electrical current. The physical therapy group underwent treatment consisting of 10 sessions of routine physical therapy modalities. The Visual Analog Scale (VAS) for pain and disability and the knee injury and osteoarthritis outcome score (KOOS) questionnaire were used to assess the outcomes before the intervention and three months after the intervention.

**Results:**

Twenty participants were analyzed in each group. The VAS for pain and disability reduced in both groups during the study; however, the difference between the two groups was not significant (P = 0.999 for pain and P = 0.873 for disability). No significant difference was observed in the KOOS symptom score either during the study or between the two groups (P = 0.423). The changes in the “pain” scores of the KOOS questionnaire were significant both in the electroacupuncture group (P = 0.003) and in the physical therapy group (P = 0.038); however, the difference was not significant between the two groups. The “activities of daily living” scores of the patients were significant both during the study and between the two groups (P = 0.042), with a steeper improvement in the electroacupuncture group. The patients’ “sports and recreational activities” scores were significant in the electroacupuncture group (P = 0.001) and between the two groups (P = 0.023). The “knee-related quality-of-life” scores were significant in both groups, and the comparison of the two groups indicated that the slope of the changes was higher in the electroacupuncture group than in the physical therapy group (P = 0.001).

**Conclusions:**

According to the results of the current research, electroacupuncture can improve the symptoms and function of patients with CMP, and its efficacy is equivalent to that of physical therapy.

## 1. Background

Chondromalacia patella (CMP) refers to the softening of the articular cartilage of the patella, often associated with fibrillation and cartilage erosions (1). This condition is typically defined by a clinical syndrome of pain in the kneecap accompanied by other symptoms such as swelling, giving way, joint crepitus, and mild quadriceps atrophy (2). The prevalence rate of this disease in the general population is reported to be 25%. The pain is intensified by vigorous physical activity, walking downhill, going up or down stairs, and prolonged sitting with bent knees (3).

In severe cases, a combination of conservative actions, including quadriceps-strengthening exercises, ice or heat modalities, patellar mobilization, and non-steroidal anti-inflammatory drugs (NSAIDs), can be helpful in treatment (4, 5). Continuous severe symptoms may necessitate surgery, although no surgical procedure has been widely successful (6). The impacts of quadriceps-strengthening exercise therapy are still controversial; however, closed-chain exercises in the terminal degrees of knee extension may improve the functioning of the patellofemoral joint by promoting the strength of the quadriceps and adjusting the patellar alignment (7, 8). Needling, along with rehabilitation, has shown superiority regarding the severity and duration of pain relief compared to pharmacotherapy plus rehabilitation (9).

Acupuncture, as a safe and effective Chinese traditional medicine, has gained higher acceptance compared to conventional therapies. Acupuncture has an analgesic effect, and electroacupuncture is more effective than manual acupuncture (5). More recent studies have indicated the release of endorphins in the cerebrospinal fluid subsequent to electroacupuncture intervention. Moreover, low-frequency (2 Hz) and high-frequency (100 Hz) electroacupuncture have been shown to selectively lead to the release of enkephalin and dynorphin in laboratory animals and humans. Clinical studies on the effectiveness of electroacupuncture in treating various musculoskeletal diseases have been debated (10).

## 2. Objectives

Given the relatively high prevalence of CMP among young soldiers and the beneficial impacts of electroacupuncture with its lower rate of complications, the present study was conducted to evaluate and compare the effectiveness of electroacupuncture versus physical therapy in the treatment of CMP. Pain and impairment of normal functioning, as the main manifestations of this disorder, were compared using the Visual Analog Scale (VAS) and knee injury and osteoarthritis outcome score (KOOS) questionnaire.

## 3. Methods

This interventional study, a randomized, single-blinded clinical trial, has been registered on Iran’s clinical trials website (ID No.: IRCT20180416039323N4). The research was conducted in the clinical biomechanics research center and the physical medicine department of AJA University of Medical Sciences at Imam Reza Hospital in Tehran in 2023. The project was reviewed and approved by the Ethics Committee of AJA University of Medical Sciences based on the submitted documents (ID No.: IR.AJAUMS.REC.1402.086) on July 17, 2023.

The inclusion criteria were soldiers diagnosed with CMP, aged 18 - 30 years, with a disease duration of more than 2 months, and lack of improvement with conservative treatments for at least one month. Chondromalacia patella was diagnosed based on the patient’s clinical symptoms, including anterior knee pain and, in some cases, posterior knee discomfort that is intensified by prolonged sitting with bent knees (theatre sign), going up and down stairs, and squatting. Exclusion criteria included a history of lower limb surgery or serious knee trauma within the last 6 months, mild trauma within the last 2 months, history of steroid drug use, coagulation problems, systemic diseases (e.g., diabetes, rheumatoid arthritis), scar at the acupuncture site, implantable cardiac defibrillators, programmable intrathecal devices, and/or disseminated cancer. Patients with evidence of intra-articular or extra-articular injury diagnosed by examination or radiography were also excluded.

Patients meeting the inclusion criteria were examined by the study researchers. Knee X-rays in frontal and lateral views were obtained to rule out any structural abnormalities. All study steps were explained to the patients, and signed written consent was obtained. One leg of each patient was included in the study, and if both legs had symptoms, the knee with more severe symptoms was included.

In the electroacupuncture group, patients lay quietly on the bed while treatment was administered at acupuncture points ST34, ST36, ST38, SP9, SP10, and GB34 using 0.3 × 30 mm sterile needles with a penetration depth of 10 mm for 20 minutes. An electroacupuncture device (Hwato Electronic Acupuncture Stimulator Sdz-II, made in China) was used to generate current with a frequency of 5 - 20 Hz and an intensity within the patient’s tolerance range, ensuring no significant muscle twitch. In the physical therapy group, patients underwent 10 sessions of routine physical therapy modalities, including infrared, ultrasound, and transcutaneous electrical nerve stimulation (TENS), for a total of 20 minutes. Both groups received 10 consecutive daily sessions. Patients were advised not to take any particular drug, but in cases of severe pain, they could take acetaminophen 325 mg once a day. Stretching exercises for the calf and hamstring muscles and strengthening exercises for the quadriceps muscles were taught to the patients in both groups.

The VAS score was used to evaluate primary outcomes such as pain and disability, with a 10 cm VAS measuring pain and disability subjectively from 0 (no pain) to 10 (most severe pain). Secondary outcomes were evaluated using the KOOS questionnaire, a self-report questionnaire covering pain, symptoms, daily activities, recreation, and quality of life. Each item is scored from zero to four, with subscales calculated on a score from zero (severe symptoms) to 100 (no symptoms). The Persian version of the KOOS questionnaire was validated in a study on 147 patients with knee problems (11).

Patients were assigned to one of the two groups using the block randomization method, ensuring equal group sizes of 22 participants each (44 patients overall). Block randomization with varying block sizes was used, and permutations for equal group sizes were randomly selected. Random numbers were generated using random allocation software version 1. The individuals explaining and recording the outcome questionnaires and the analyst were blinded to patient allocation.

Data analysis was performed using SPSS software version 26. Results were reported as mean and standard deviation (mean ± SD). The Kolmogorov-Smirnov test assessed the normal distribution of each group of data. The independent *t*-test compared the means between the two groups, while repeated measures ANOVA compared mean repetitions over time. Mixed ANOVA was also used to compare these repetitions between groups.

## 4. Results

**Figure 1.**
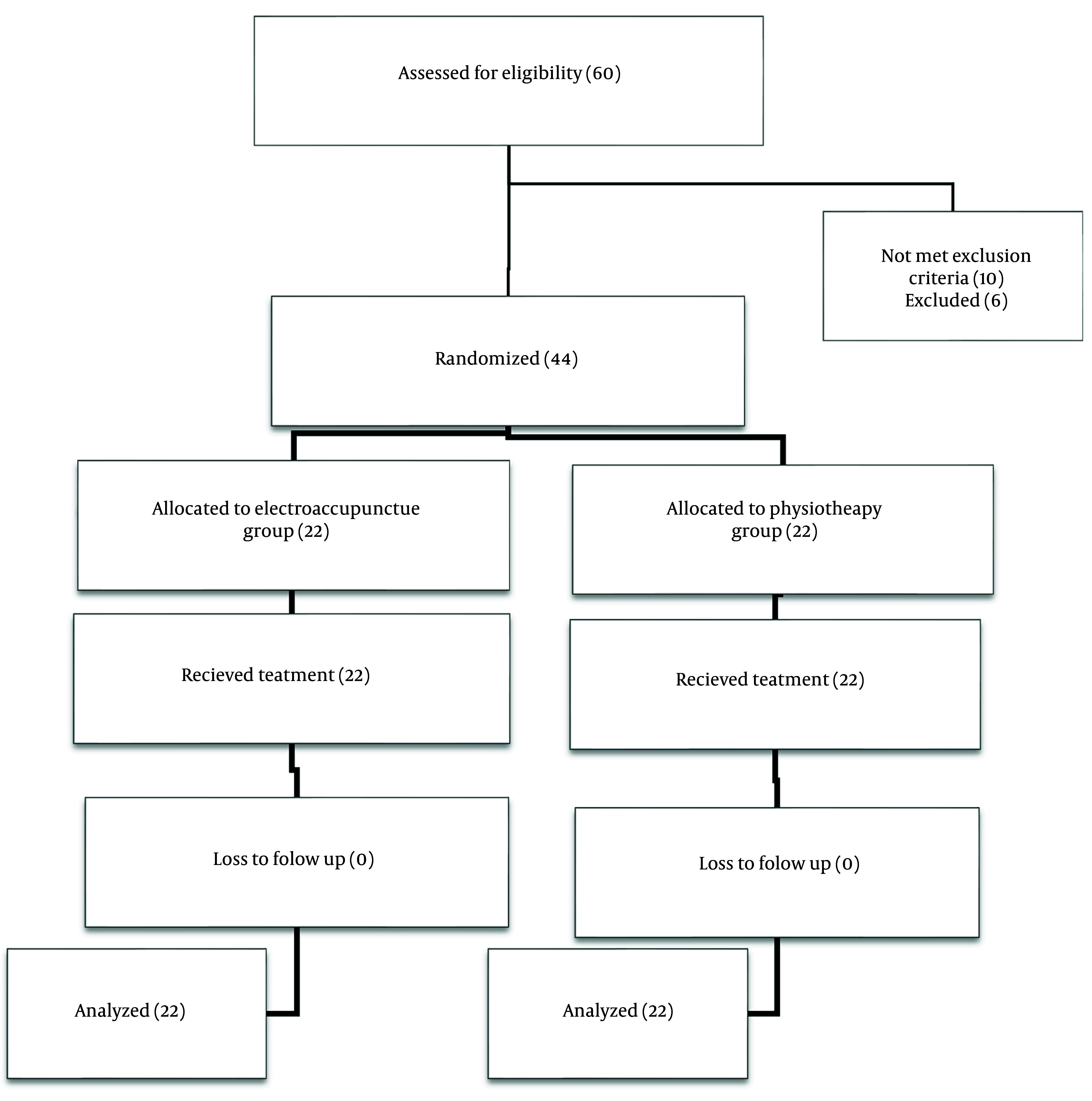
Patients’ flow diagram

This study was completed with 22 patients in each group (44 patients overall), with no dropouts (Figure 1). Table 1 shows the comparison of the basic characteristics of patients in the two groups. No significant differences were observed between the two groups in the main variables, such as pain VAS (*t*(38) = 0.751, P = 0.457, d = 0.238), disability VAS (*t*(38) = 0.788, P = 0.583, d = 0.247), and the KOOS total score (*t*(38) = 0.916, P = 0.366, d = 0.960). The analysis also indicated that age (F(1,37) = 0.410, P = 0.526) and body mass index (BMI) (F(1,37) = 0.837, P = 0.366) had no effect on the patient’s initial pain VAS level.

**Table 1.** Baseline Characteristics of Patients

Features	Electroacupuncture (20)	Physiotherapy (20)	P-Value
**Age (y)**	24.8 ± 6.7	25.4 ± 5.4	0.616
**BMI**	26.1 ± 5.8	27.8 ± 6.7	0.232
**VAS**			
Pain	6.40 ± 1.31	6.10 ± 1.21	0.457
Disability	6.25 ± 1.41	6.60 ± 1.53	0.583
**KOOS**			
Symptoms	64.64 ± 17.14	68.92 ± 14.52	0.399
Pain	54.16 ± 23.98	56.72 ± 11.27	0.675
ADL	57.89 ± 23.14	61.25 ± 16.62	0.605
Sports	46.25 ± 26.05	55.52 ± 24.03	0.256
Quality of life	33.55 ± 16.43	40.00 ± 14.53	0.202
Total	51.35 ± 15.47	55.73 ± 13.07	0.366

Abbreviations: BMI, body mass index; VAS, Visual Analog Scale; KOOS, knee injury and osteoarthritis outcome score.

Table 2 shows the pain and disability VAS and the KOOS questionnaire scores before the intervention and three months after the intervention. The pain VAS score reduced significantly in both groups; however, no significant difference was observed in pain VAS reduction between the two groups. Additionally, the KOOS pain subgroup also showed a significant reduction similar to the pain VAS score, and this reduction was equal in both groups. The disability VAS scores decreased significantly in both groups, with no significant difference between the two groups. Based on the KOOS score, there was no significant difference in the symptoms of the patients between the two groups. Both the “activities of daily living” and “quality of life” scales improved significantly in both groups, with significant differences between the two groups. The changes in the scores of patients' “sports and recreational activities” were significant only in the electroacupuncture group, but the difference was significant between the two groups. Ultimately, in the trend of changes in the KOOS total score, improvement was observed only in the electroacupuncture group; these changes were not significant between the two groups either.

**Table 2.** Between- and Within-Group Analyses for Change in Pain Visual Analog Scale, Disability Visual Analog Scale and Knee Injury and Osteoarthritis Outcome Score Subscales ^a^

Features	Electroacupuncture	Physiotherapy	*t*-Statistic ± DoF	P-Value	Effect Size ± Cohens D
**VAS**					
**Pain**					
Baseline - 3 months	1.60 ± 0.88; [1.18, 2.01]	1.55 ± 0.82; [1.16, 1.93]	0.185 ± 38	0.854	0.059
*t*-statistic (DoF)	8.10 ± 19	8.39 ± 19			
P-value	< 0.001 ^b^	< 0.001 ^b^			
Effect size (partial eta square)	0.776	0.788			
**Disability**					
Baseline - 3 months	0.90 ± 0.85; [0.50, 1.29]	0.95 ± 1.09; [0.43, 1.46]	-0.161 ± 38	0.873	-0.051
*t*-statistic (DoF)	4.72 ± 19	3.86 ± 19			
P-value	< 0.001 ^b^	0.001 ^b^			
Effect size	1.05	0.864			
**KOOS**					
**Symptoms**					
Baseline - 3 months	2.50 ± 9.05; [-1.73, 6.73]	-2.50 ± 26.09; [-14.71, 9.71]	0.810 ± 38	0.423	0.256
*t*-statistic (DoF)	1.23 ± 19	-4.28 ± 19			
P-value	0.232	0.673			
Effect size	0.276	0.096			
**Pain**					
Baseline - 3 months	-8.05 ± 10.58; [-13.00, -3.10]	-2.3 ± 5.11; [-5.09, -0.16]	-2.020 ± 38	0.051	-0.647
*t*-statistic (DoF)	-3.40 ± 19	-2.24 ± 19			
P-value	0.003 ^b^	0.038 ^b^			
Effect size	0.761	0.515			
**ADL**					
Baseline - 3 months	-8.20 ± 13.79; [-14.85, -1.55]	-1.54 ± 3.14; [-3.01, -0.71]	-2.052 ± 19.7	0.042 ^b^	-0.107
*t*-statistic (DoF)	-2.59 ± 19	-2.59 ± 19			
P-value	0.018 ^b^	0.041 ^b^			
Effect size	0.595	0.491			
**Sports**					
Baseline - 3 months	-8.00 ± 8.94; [-12.18, -3.81]	-1.62 ± 7.75; [-5.35, 2.11]	-2.378 ± 38	0.023 ^b^	-0.761
*t*-statistic (DoF)	-4.10 ± 19	-0.91 ± 19			
P-value	0.001 ^b^	0.374			
Effect size	0.894	0.209			
**Quality of life**					
Baseline - 3 months	-4.60 ± 7.16; [-8.06, -1.14]	3.43 ± 6.86; [0.22, 6.65]	-3.578 ± 38	< 0.001 ^b^	-1.146
*t*-statistic (DoF)	-2.80 ± 19	2.23 ± 19			
P-value	0.012 ^b^	0.037 ^b^			
Effect size	0.642	0.500			
**Total**					
Baseline - 3 months	-5.76 ± 8.18; [-9.83, -1.69]	-1.01 ± 6.26; [-4.12, 2.10]	-1.954 ± 38	0.059	-0.651
*t*-statistic (DoF)	-2.98 ± 19	-0.68 ± 19			
P-value	0.008 ^b^	0.501			
Effect size	0.704	0.162			

Abbreviations: DoF, degrees of freedom; VAS, Visual Analog Scale; KOOS, knee injury and osteoarthritis outcome score.

^a^ Values are expressed as mean ± SD; [95% CI] unless otherwise indicated.

^b^ Significant at the level of 0.05.

**Figure 2.**
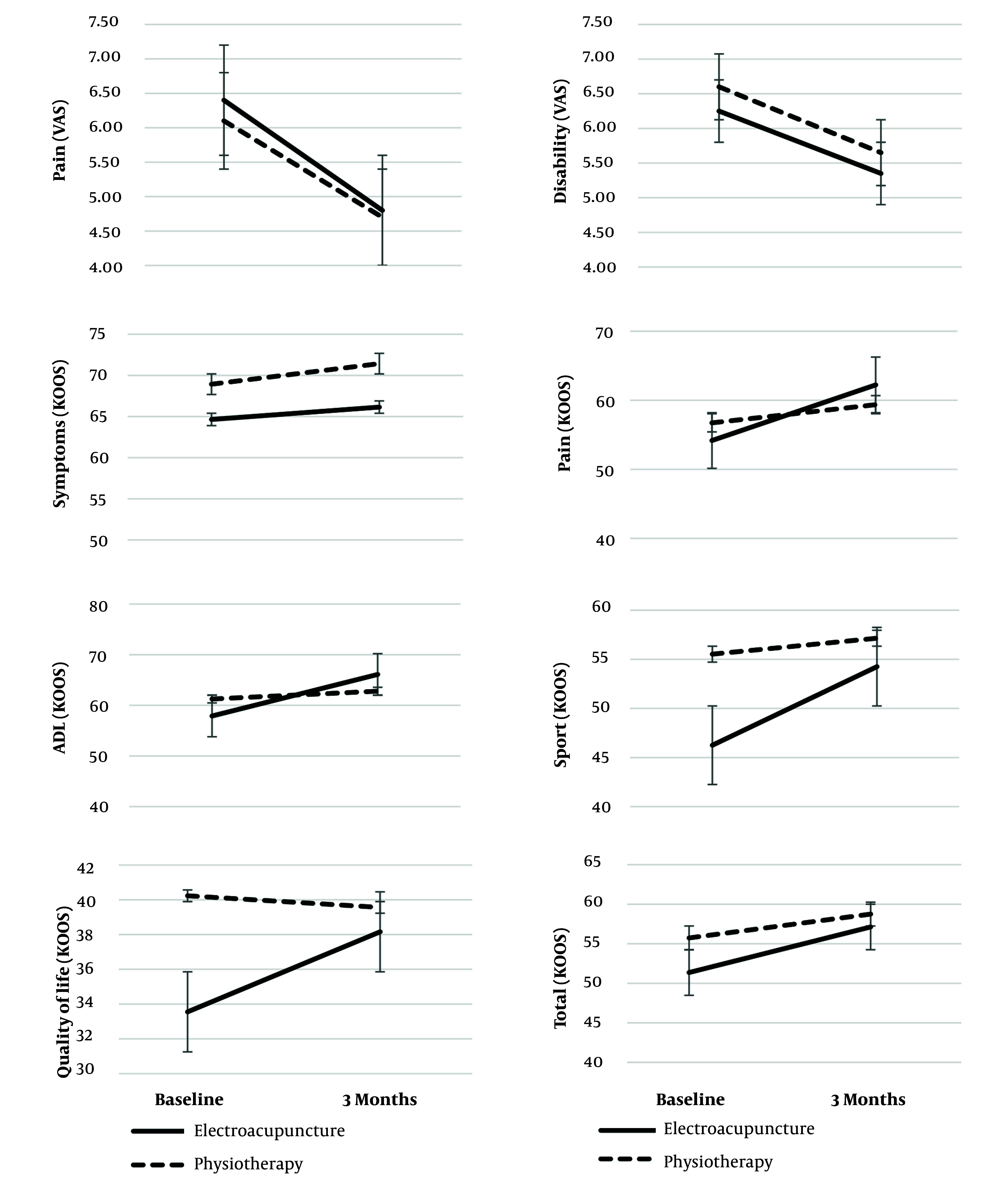
Changes of the mean outcomes throughout the study for electroacupuncture (20) and physiotherapy (20) group. Error bars represent the 95% CI for the outcomes.

Figure 2 also evaluates the changes in the investigated variables. The degree of pain and disability relief based on VAS was similar in patients in both groups. Also, among the subgroups of the KOOS questionnaire, the degree of changes in sports activities and quality of life showed a greater slope in the electroacupuncture group than in the physiotherapy group. The two groups had no significant differences in the slope of the KOOS total scores.

## 5. Discussion

In this study, we investigated the effectiveness of electroacupuncture compared to physiotherapy in relieving pain and disability in soldiers with CMP. Although there are extremely limited studies on the effectiveness of electroacupuncture in the treatment of CMP, a review study by Lv et al. (5) shows that acupuncture can significantly increase clinical effectiveness and reduce pain scores compared to NSAIDs. No studies have reported acupuncture-related complications. Moreover, based on the findings of the mentioned review study, acupuncture therapy has been more effective in improving clinical effectiveness and pain scores compared to NSAIDs.

While this study examined acupuncture without electrical stimulation, it demonstrated that acupuncture improved the pain VAS score in patients with CMP compared to NSAIDs, which is consistent with our findings. Furthermore, the results of Haghighat’s study (12) indicated that although the pain VAS reduced significantly in both electroacupuncture and sham electroacupuncture groups, the descending trend was similar between the two groups. In another case study by Tootill et al. (13), it was found that after 15 weeks, the patient’s usual pain VAS decreased from 6 to 1, and the patient’s worst pain VAS decreased from 7.6 to 3.5, which was a significant reduction. In our study, we also used the disability VAS, and according to the results, the degree of disability significantly reduced in both electroacupuncture and physiotherapy groups; however, there was no significant difference between the two groups.

Although Haghighat et al.’s study (12) used the Anterior Knee Pain Scale (AKPS) criterion to evaluate pain and functioning in patients with CMP, the results demonstrated that the patellofemoral syndrome score improved significantly in the electroacupuncture group; however, this rate was not significantly different in the sham electroacupuncture group, and finally, there was no significant difference between the two groups. In Tootill et al.’s study (13), similar to our study, the KOOS criterion was used. The results, consistent with our study, showed that after 15 weeks, the “pain,” “daily life functioning,” “functioning, sports, and recreational activities,” and “quality of life” scores in the KOOS questionnaire reduced, but the patients’ symptom scores did not significantly reduce.

Other studies have explored different methods for treating CMP. Qiu et al. (9) assessed the effectiveness of a warming needle compared to rehabilitation in the treatment of CMP and showed that a warming needle, along with rehabilitation, had a greater therapeutic effect and a longer duration of relief than pharmacotherapy combined with rehabilitation training in the treatment of CMP.

The effects of physiotherapy in the treatment of CMP have been investigated in numerous studies. In a review study, Lake and Wofford (14) revealed that therapeutic methods such as ultrasound, cold, phonophoresis, iontophoresis, neuromuscular electrical stimulation, electrical stimulation for pain control, electromyographic biofeedback, and laser, in combination with other treatments, might be useful for controlling pain or other symptoms. When used alone in the treatment of CMP, there is no stable evidence of the usefulness of these methods. In our study, multiple physiotherapy modalities, such as superficial and deep heat and electrical stimulation, were used concurrently for patients. Although these modalities significantly reduced pain and disability VAS scores and improved the pain, quality of life, and daily life functioning subscales of the KOOS questionnaire, there was no significant difference between the two groups in the measured criteria.

### 5.1. Conclusions

According to the results of the current study, electroacupuncture can relieve pain and improve functioning in patients with CMP, leading to better pain and disability VAS scores as well as improved KOOS scores. However, the improvement in these criteria is similar to that achieved with physiotherapy, indicating that electroacupuncture can have effects comparable to physiotherapy. Given the shorter duration and the need for fewer facilities, electroacupuncture can be considered an alternative treatment for physiotherapy in patients with CMP.

## Data Availability

The dataset presented in the study is available on request from the corresponding author during submission or after publication.

## Clinical Trial Registration Code:

This interventional study, as a randomized, single-blinded clinical trial, has been registered on Iran’s clinical trials website (ID No.: IRCT20180416039323N4).
